# *Toxoplasma gondii* Infections in Animals and Humans in Southern Africa: A Systematic Review and Meta-Analysis

**DOI:** 10.3390/pathogens11020183

**Published:** 2022-01-28

**Authors:** Adejumoke O. Omonijo, Chester Kalinda, Samson Mukaratirwa

**Affiliations:** 1 Department of Animal and Environmental Biology, Faculty of Science, Federal University Oye, Oye-Ekiti 370112, Nigeria; 2 School of Life Sciences, College of Agriculture, Engineering and Science, Westville Campus, University of KwaZulu-Natal, Durban 4001, South Africa; mukaratirwa@ukzn.ac.za; 3 Bill and Joyce Cummings Institute of Global Health, University of Global Health Equity (UGHE), Kigali Heights, KG 7 Ave., Kigali P.O. Box 6955, Rwanda; ckalinda@gmail.com; 4 Howard College Campus, School of Nursing and Public Health, College of Health Sciences, University of KwaZulu-Natal, Durban 4001, South Africa; 5 Institute of Global Health Equity Research (IGHER), University of Global Health Equity (UGHE), Kigali Heights, KG 7 Ave., Kigali P.O. Box 6955, Rwanda; 6 One Health Center for Zoonoses and Tropical and Veterinary Medicine, Ross University School of Veterinary Medicine, Basseterre KN0101, Saint Kitts and Nevis

**Keywords:** *Toxoplasma gondii*, infections, southern Africa, systematic review, animals, humans

## Abstract

Background: *Toxoplasma gondii* is an apicomplexan parasite with zoonotic importance worldwide especially in pregnant women and immunocompromised people. This study is set to review the literature on *T. gondii* infections in humans and animals in southern Africa. Methods: We extracted data regarding *T.* *gondii* infections from published articles from southern Africa from 1955 to 2020 from four databases, namely Google Scholar, PubMed, EBSCO Host, and Science Direct. Forty articles from eight southern African countries were found eligible for the study. Results: This review revealed a paucity of information on *T*. *gondii* infection in southern African countries, with an overall prevalence of 17% (95% CI: 7–29%). Domestic felids had a prevalence of 29% (95% CI: 7–54%), wild felids 79% (95% CI: 60–94), canids (domestic and wild) 69% (95% CI: 38–96%), cattle 20% (95% CI: 5–39%), pigs 13% (95% CI: 1–29%), small ruminants (goats and sheep) 11% (95% CI: 0–31%), chicken and birds 22% (95% CI: 0–84%), and humans 14% (95% CI: 5–25%). Enzyme-linked immunosorbent assay (ELISA) and immunofluorescence antibody test (IFAT) constituted the most frequently used diagnostic tests for *T. gondii*. Conclusions: We recommend more focused studies be conducted on the epidemiology of *T*. *gondii* in the environment, food animals and human population, most especially the at-risk populations.

## 1. Introduction

*Toxoplasma gondii* is an apicomplexan obligate parasite that infects animals and humans worldwide [1]. The definitive hosts are felids although a recent study showed developmental success in mice subjected to certain enzymatic inhibition and diet modification [2]. The intermediate hosts include terrestrial and aquatic mammals and birds [2,3]. The pathways of *T. gondii* infection and transmission are multifaceted, involving the three developmental stages (tachyzoite, bradyzoite, and sporozoite) of the parasite’s life cycle [2]. Intermediate hosts, including humans, can acquire infection via (i) consumption of water, vegetables, and fruits contaminated with infective oocysts; (ii) consumption of raw or undercooked meat infected with tachyzoites or bradyzoites [4]; (iii) blood transfusion; (iv) organ transplant containing cysts or tachyzoites; and (v) congenital transmission from the mother to fetus via the placenta. Feline definitive hosts acquire infections via the ingestion of sporulated oocysts or by carnivorism. However, rarely, consumption of non-pasteurized milk or milk products can serve as a potential source of *T*. *gondii* transmission [2,5,6]. Oysters and mussels can act as reservoir hosts for infective oocysts, which can later be transmitted to other animals upon consumption [2,7,8,9]. Parasites attain maturity in the intestine of felids and start releasing numerous oocysts into the environment within three to 18 days post-infection [10].

Furthermore, *Toxoplasma* infection in animals or humans causes toxoplasmosis which is prevalent worldwide. The infection rate varies according to geographic region and climatic conditions [1]. Other risk factors of infection include age, gender, farm management, and geographic characteristics [5]. Toxoplasmosis is accompanied by varying degrees of clinical symptoms depending on the inoculum size, virulence of parasite strain, and level of host immunity [11]. *Toxoplasma* infections have been reported to alter reproductive parameters in hosts by having a negative impact on harming female reproductive functions [12], inducing apoptosis in spermatogonial cells directly or indirectly [13], thereby resulting in reduced quality of human sperm [14] and decreased fertility in experimentally infected male rats [13,15]. A significant association has been reported between *T. gondii* seropositivity and abortion in small ruminants from certain districts of central Ethiopia [16]. In sheep, an infection may cause early embryonic death and resorption, fetal death and mummification, abortion, and stillbirth, [17] thereby resulting in severe economic loss in the livestock industry [1,3]. The economic impact of *T. gondii* infection in sheep and other livestock is abortions and increased lambing/kidding interval, culling of infected animals, reduced milk production, and reduced value of the breeding stock, hence leading to major economic losses [16]. The severity of infection is dependent on the stage of gestation the ewe acquires infections. Infection at the early gestational stage often results in fatal consequences [16,18]. In immunocompetent hosts, toxoplasmosis may be asymptomatic, whereas in immunocompromised humans, particularly AIDS patients, the disease has serious consequences [3,19]. Similarly, infection in pregnant women is associated with congenital toxoplasmosis, and the severity and risk are dependent on the time of maternal infection and often accompanied by developmental malformation, abortion, or reduced quality of life for the child [3,11,19].

While toxoplasmosis is a zoonosis that can be controlled or prevented in humans and animals worldwide, in sub-Saharan Africa, the control is hampered by various factors, including high poverty level, lack of diagnostic capacity, limited disease surveillance, and poor veterinary care [20]. Since the fecal-oral route and consumption of raw or undercooked infected food or meat constitute the major transmission route in humans [11], effective control of toxoplasmosis requires adequate awareness of good veterinary practices, personal hygiene, improved culinary habits, dietary habits, and correct diagnosis.

Diagnosis involves direct methods, immunodiagnostic methods, and molecular techniques. The direct method involves isolation of parasite or bioassay, cellular culture, and histology. Immunodiagnostic methods include the Sabin–Feldman dye test (SFT), hemagglutination assay, immunofluorescent assay (IFA), modified agglutination test (MAT), avidity, western blot, enzyme-linked immunosorbent assay (ELISA), recombinant antigens, immunocytochemistry, and immunohistochemistry. Molecular techniques include Polymerase Chain Reaction (PCR), real-time PCR, PCR-restriction fragment length polymorphisms (PCR-RFLP), loop-mediated isothermal amplification (LAMP), and high-resolution melting (HRM) [21].

*Toxoplasma gondii* infection is accompanied by the emergence of IgM in the host, followed by the appearance of IgA and IgE at about two weeks post-infection [22,23] while IgG spikes around four months post-infection and persists throughout lifetime [23]. Toxoplasmosis in immunocompetent individuals resolves without treatment [24], but in immunocompromised individuals, clindamycin, sulfonamides, spiramycin, and pyrimethamine are used for treatment [25,26]. Pyrimethamine and sulfadiazine drug combination is suitable for new-borns, infants, and pregnant women; however, to prevent transmission from mother to unborn fetus, an antibiotic (spiramycine) has been proven effective but not in latent infections, as antibiotics are unable to reach the bradyzoites in adequate concentrations [23,27].

Toxoplasmosis prevention is centered around avoidance of contact with sources of infection, such as cats, contaminated environment, consumption of raw or undercooked meat, personal hygiene, and regular handwashing [23]. The control of mechanical vectors of transmission, such as cockroaches, flies, or rodents in the surroundings, can also be adopted in disease control [24]. This review aims to analyze published literature on *Toxoplasma* infections in animals and humans in southern Africa and determine the epidemiological distribution of infection in various hosts in the region and identify gaps for future research.

## 2. Results

### 2.1. Systematic Review

**Figure 1:**
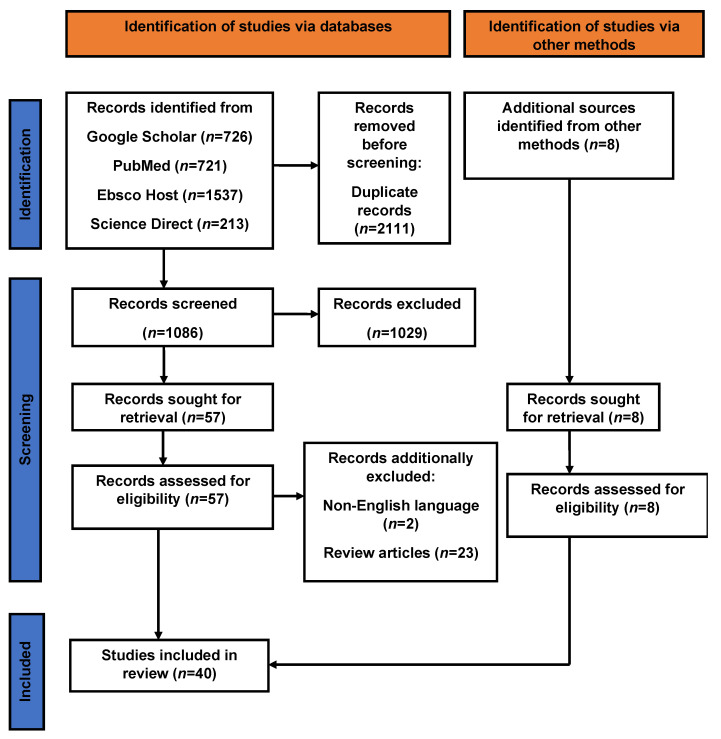
PRISMA.

A total of 3197 articles were identified from the following databases: Google Scholar, PubMed, EBSCO Host, and Science Direct. After duplicates (*n* = 2111) were removed, title and abstracts were perused for 1086 articles. An additional eight studies were identified from other sources. Overall, 1029 articles were excluded because they were not original articles, non-relevant to research objectives to the study, or abstracts. Of the 65 reviewed full-text articles, 40 were selected for inclusion in the systematic and meta-analysis. A flow diagram illustrating this selection process is presented in Figure 1.

### 2.2. Quality Assessment of Articles and Diagnostic Tests Used

The quality index of the reviewed articles ranged from 0.4 to 0.9. Diagnostic tests used in detecting the presence of *T. gondii* in the studies are shown in Table 1, Table 2, Table 3, Table 4 and Table 5. Sample size ranged from 1–159 for domestic felids (Table 1), 1–250 for wild felids (Table 2), 4–39 for canids (Table 1), 109–184 for cattle (Table 3), 128–156 for goats (Table 3), 121–600 for sheep (Table 3), 70–311 pigs (Table 3), 16–137 for chicken and birds (Table 4), 20 for blue wildebeest (Table 2), 90 for baboons (Table 2), 20 for springbok (Table 2), and 1–3379 for humans (Table 5).

### 2.3. Results from the Meta-Analysis

#### 2.3.1. Pooled Prevalence and Heterogeneity

**Figure 2:**
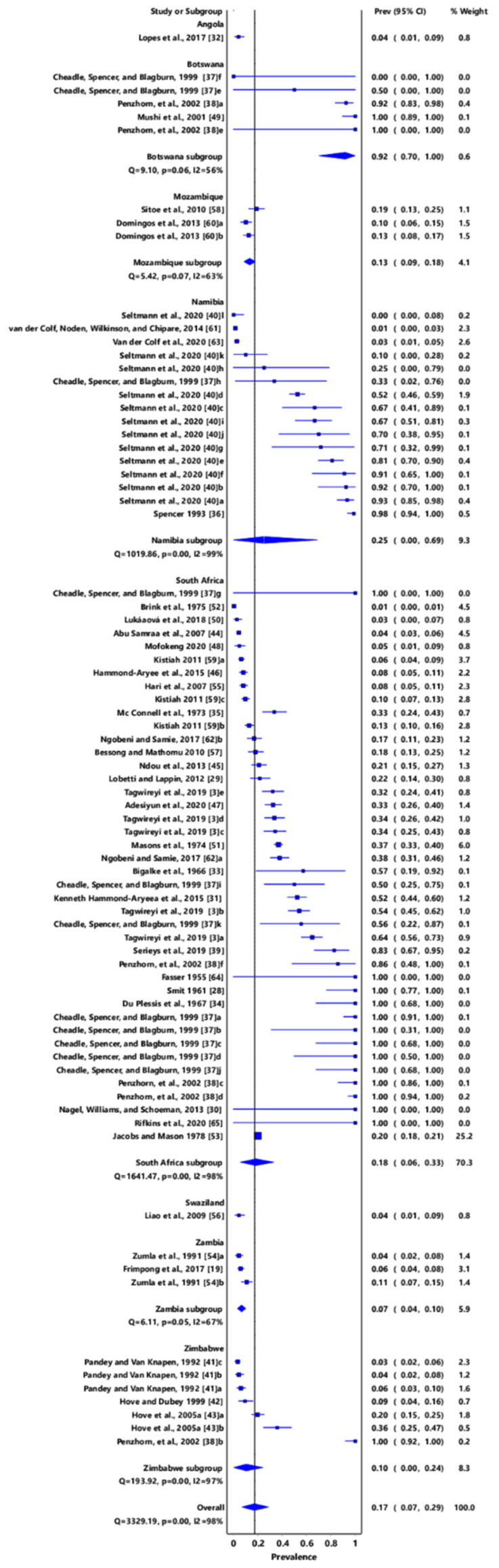
Forest plot of prevalence estimates of *Toxoplasma gondii* infections in Southern Africa. The confidence interval (CI) was 95%, and the diamond represents the pooled estimate (blue squares represent point estimation of the study weighted for population size) [3,19,28,29,30,31,32,33,34,35,36,37,38,39,40,41,42,43,44,45,46,47,48,49,50,51,52,53,54,55,56,57,58,59,60,61,62,63,64,65].

*Toxoplasma gondii* infection in southern African countries had an overall prevalence of 17% (95% confidence interval (CI): 7–29%). Angola had a prevalence of 4% (95% CI: 1–9%); Botswana, 92% (95% CI: 70–100%); Mozambique, 13% (95% CI: 9–18%); Namibia, 25% (95% CI: 0–69%); South Africa, 18% (95% CI: 6–33%); Swaziland, 4% (95% CI: 1–9); Zambia, 7% (95% CI: 4–10%); and Zimbabwe, 10% (95% CI: 0–24%) (Figure 2).

**Figure 3:**
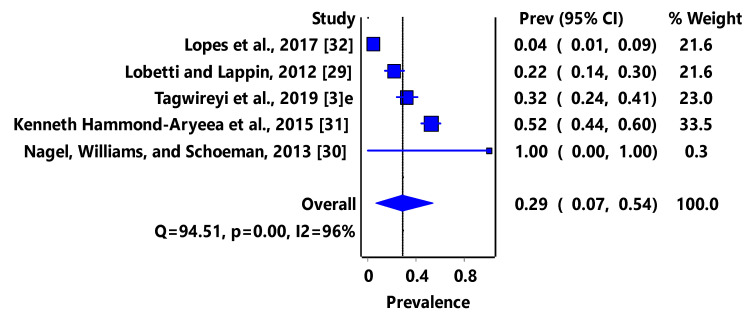
Forest plot of prevalence estimates of *Toxoplasma gondii* infections in domestic felids in southern Africa. The confidence interval (CI) was 95%, and the diamond represents the pooled estimate (blue squares represent point estimation of the study weighted for population size) [3,29,30,31,32].

**Figure 4:**
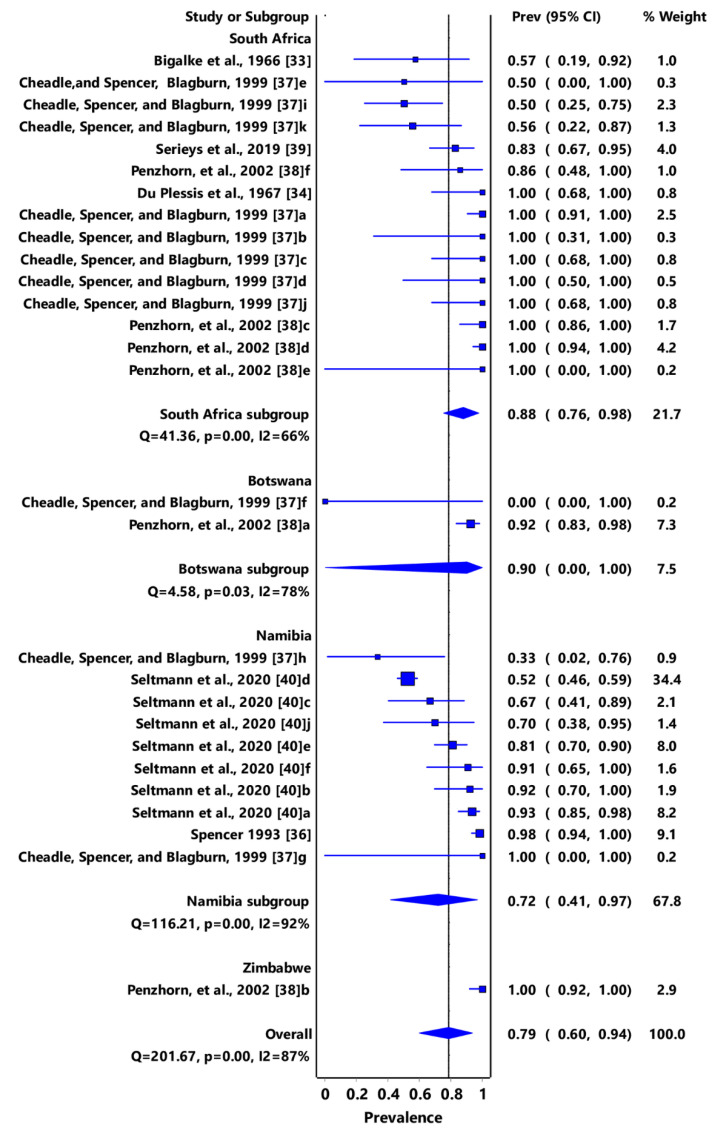
Forest plot of prevalence estimates of *Toxoplasma gondii* infections in wild felids in southern Africa. The confidence interval (CI) was 95%, and the diamond represents the pooled estimate (blue squares represent point estimation of the study weighted for population size) [33,34,36,37,38,39,40].

**Figure 5:**
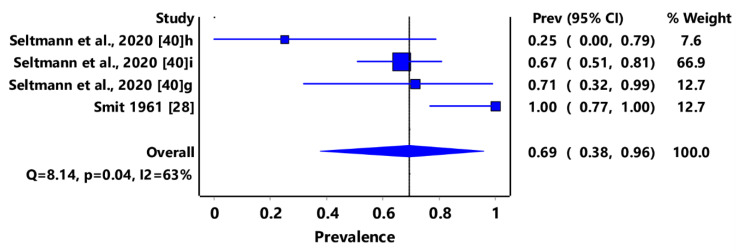
Forest plot of prevalence estimates of *Toxoplasma gondii* infections in canids in southern Africa. The confidence interval (CI) was 95%, and the diamond represents the pooled estimate (blue squares represent point estimation of the study weighted for population size) [28,40].

**Figure 6:**
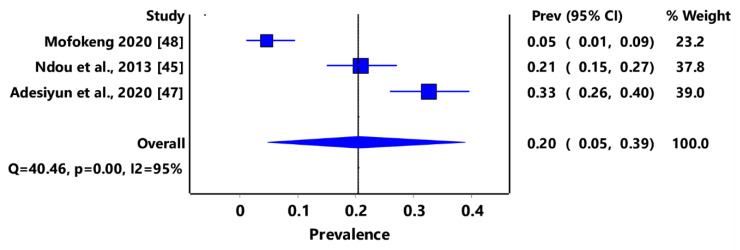
Forest plot of prevalence estimates of *Toxoplasma gondii* infections in cattle in southern Africa. The confidence interval (CI) was 95%, and the diamond represents the pooled estimate (blue squares represent point estimation of the study weighted for population size) [45,47,48].

**Figure 7:**
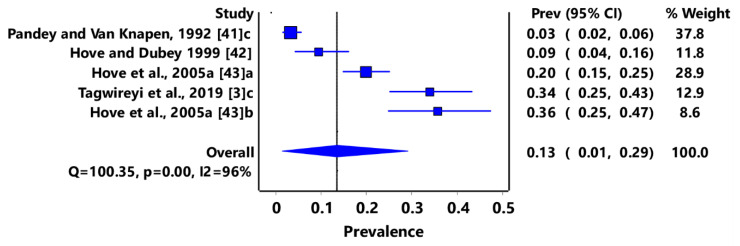
Forest plot of prevalence estimates of *Toxoplasma gondii* infections in pigs in southern Africa. The confidence interval (CI) was 95%, and the diamond represents the pooled estimate (blue squares represent point estimation of the study weighted for population size) [3,41,42,43].

**Figure 8:**
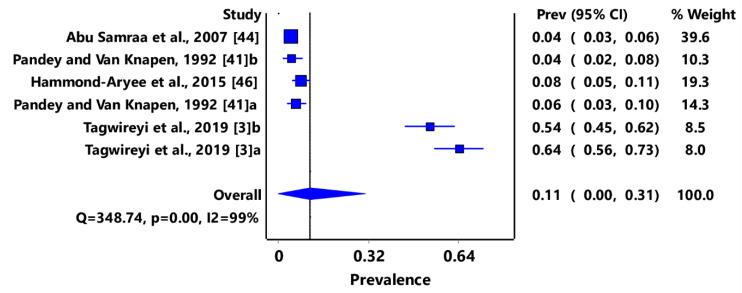
Forest plot of prevalence estimates of *Toxoplasma gondii* infections in small ruminants (sheep and goats) in southern Africa. The confidence interval (CI) was 95%, and the diamond represents the pooled estimate (blue squares represent point estimation of the study weighted for population size) [3,41,44,46].

**Figure 9:**
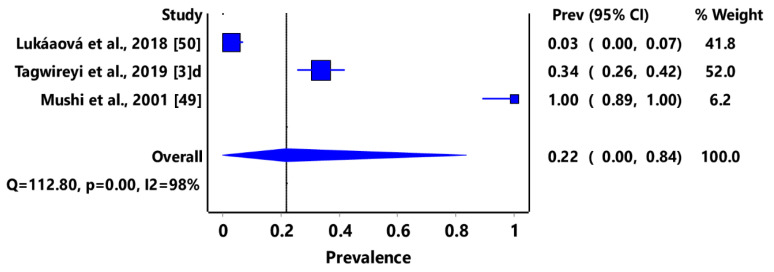
Forest plot of prevalence estimates of *Toxoplasma gondii* infections in birds in southern Africa. The confidence interval (CI) was 95%, and the diamond represents the pooled estimate (blue squares represent point estimation of the study weighted for population size) [3,49,50].

Based on animal groups, *T*. *gondii* infection in domestic felids in the region had an overall prevalence of 29% (95% CI: 7–54%) (Figure 3) and in wild felids, 79% (95% CI: 60–94%) (Figure 4). Canids (domestic and wild) had an overall prevalence of 69% (95% CI: 38–96%) (Figure 5); cattle, 20% (95% CI: 5–39%) (Figure 6); pigs, 13% (95% CI: 1–29%) (Figure 7); small ruminants (goats and sheep), 11% (95% CI: 0–31%) (Figure 8); and chicken and birds, 22% (95% CI: 0–84%) (Figure 9). The summary of studies on the prevalence of *T. gondii* in felids, canids, wildlife, livestock, and fowls in southern Africa are shown in Table 1, Table 2, Table 3 and Table 4, respectively.

#### 2.3.2. *Toxoplasma gondii* Infections in Humans in Southern African Countries

**Figure 10:**
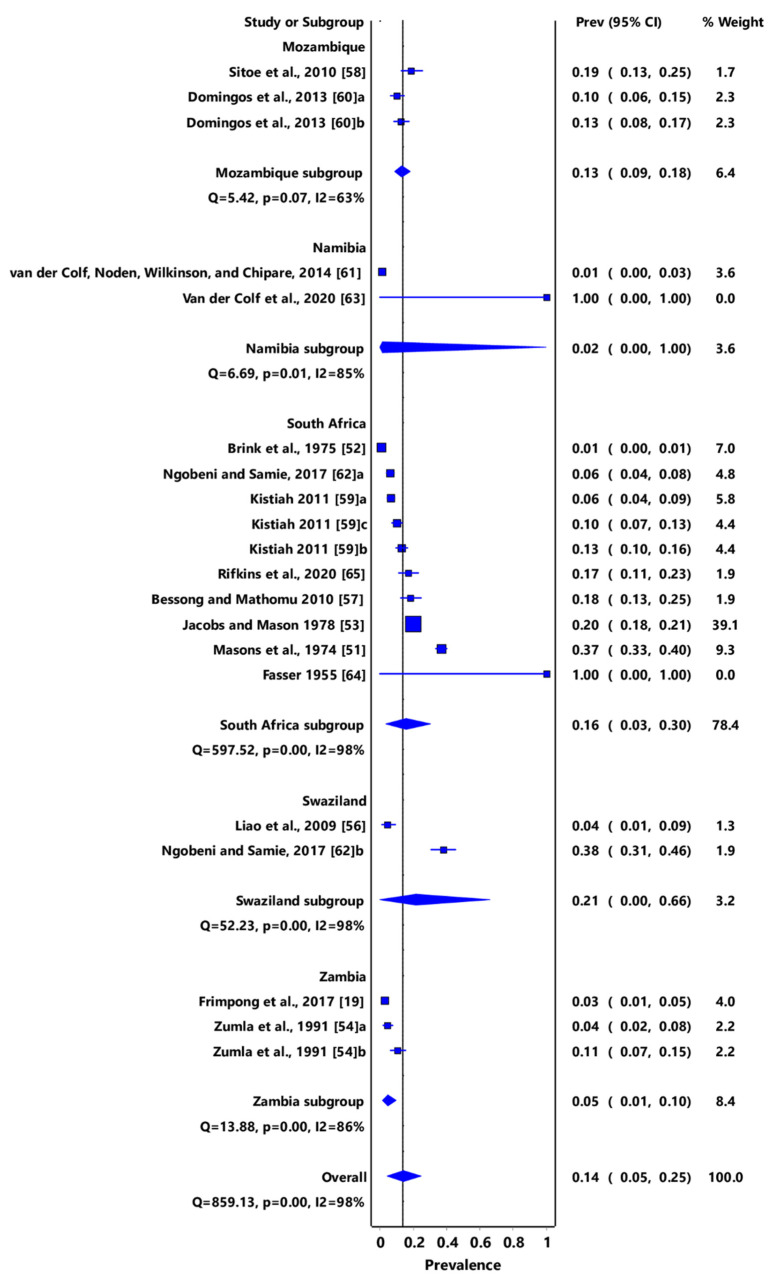
Forest plot of prevalence estimates of *Toxoplasma gondii* infections in humans in southern Africa. The confidence interval (CI) was 95%, and the diamond represents the pooled estimate (blue squares represent point estimation of the study weighted for population size) [19,51,52,53,54,56,57,58,59,60,61,62,63,64,65].

The pooled prevalence of *T. gondii* infection in humans was 14% (95% CI: 5–25%), with the highest prevalence of 17% (95% CI: 4–33%) recorded in South Africa and the least prevalence of 2% (95% CI: 1–3%) from Namibia (Figure 10). A summary of studies on *Toxoplasma* infections in humans in southern African countries is shown in Table 5. Out of a total of 8623 serum samples that were examined, 1342 were positive for *Toxoplasma* serology. Furthermore, an additional archaeological study on dead human remains was reportedly positive for *T*. *gondii*.

#### 2.3.3. Pooled Prevalence and Heterogeneity of Diagnostic Tests

**Figure 11:**
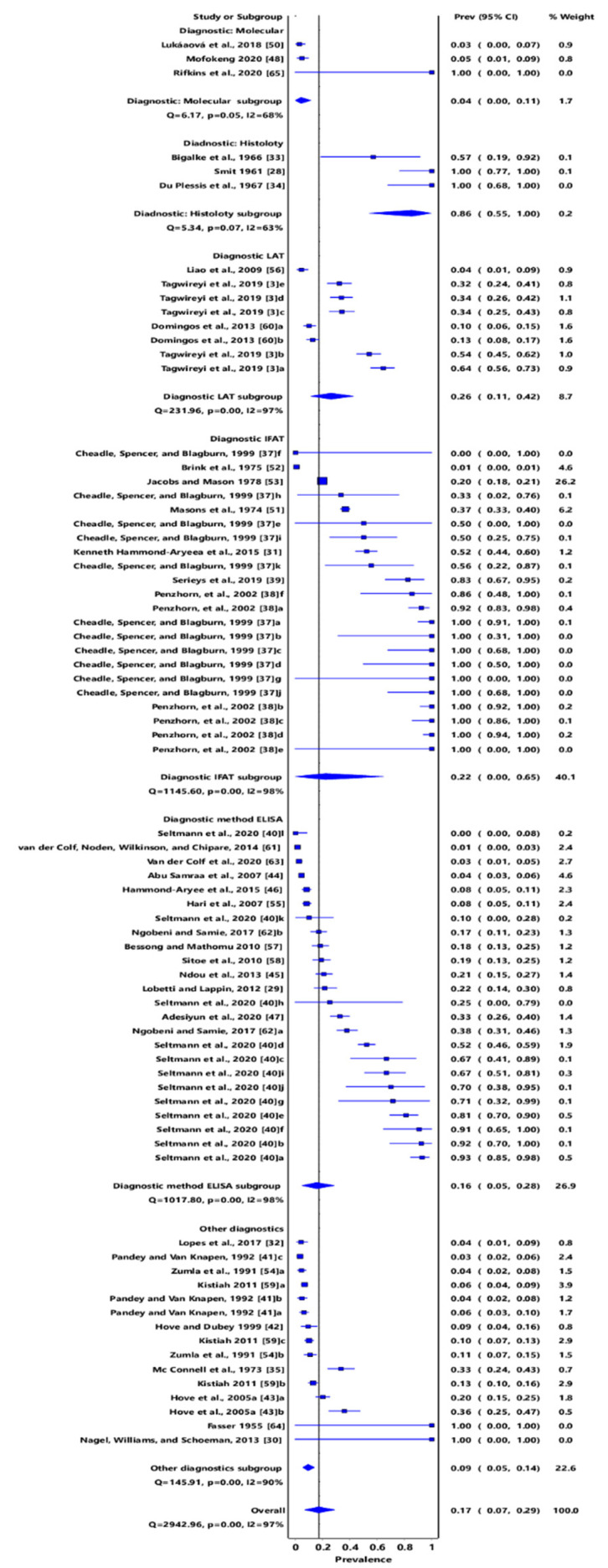
Forest plot of diagnostic methods of *Toxoplasma gondii* infections in southern Africa. The confidence interval (CI) was 95%, and the diamond represents the pooled estimate (blue squares represent point estimation of the study weighted for population size) [3,28,29,30,32,33,34,35,37,38,39,40,41,42,43,44,45,46,47,48,50,51,54,55,56,57,58,59,60,61,62,63,64,65].

Meta-analysis of the diagnostic methods used in detecting *T*. *gondii* infections in southern African countries had an overall pooled prevalence of 17% (95% CI: 7–29%). Molecular sub-group showed an estimated prevalence of 4% (95% CI: 0–11%); histology, 86% (95% CI: 55–100%); the latex agglutination test (LAT), 26% (95% CI: 11–42%); ELISA, 16% (95% CI: 5–28%); and IFAT, 22% (95% CI: 0–65%) (Figure 11). Diagnostics tests that were used less frequently, i.e., in less than three studies, were grouped separately and had a pooled prevalence of 9% (95% CI: 5–14%). These include MAT, LAT and ELISA; LAT and the Methylene blue dye test (DT); IFAT and ELISA; Pastorex Toxo latex particle agglutination test and BioMèrieux Toxo Screen DA test; and a combination of IFAT, CF (complement-fixation test), Wolstenholme’s modification, and Sabin–Feldman dye test (Figure 11).

## 3. Discussion

*Toxoplasma gondii* is a coccidian cosmopolitan parasite of global economic and zoonotic importance. The importance of *T*. *gondii* in the meat industry and public health has been reported in a wide variety of hosts and humans, especially among immunocompromised individuals. This review revealed that there is limited information on the distribution of *T*. *gondii* in animals and humans in southern African countries. In this study, the overall pooled prevalence is estimated as 17% (95% CI: 7–29%).

The overall pooled prevalence of *T*. *gondii* infection 29% (95% CI: 7–54%) in domestic felids observed in this study is lower than the pooled seroprevalence of 51% (20–81%) reported in Africa, 52% (15–89%) in Australia [10], and 30–40% global prevalence from previous studies [66,67]. However, the pooled prevalence of *T*. *gondii* infections observed in wild felids 79% (95% CI: 60–94%) in this study is higher than the pooled prevalence reported in Africa, Asia, Europe, and South America [10], while in north African countries, no data were available on wild felids [68]. The role of felids (domestic and wild) in *T*. *gondii* epidemiology has been documented in several reports [10,69,70]. In this review, seven (7) studies were on wild felids, while five (5) studies were on domestic cats. A single infected felid is capable of shedding millions of oocysts for 10–15 days, thereby contaminating the environment and posing infection risk to various intermediate hosts [70]. Emphasis on the adequate veterinary care of animals, including frequent treatment of cats for toxoplasmosis and reduction in the population of stray cats in the environment, should be encouraged in southern African countries. Moreover, a surveillance system for *Toxoplasma* infection should be instituted at the wildlife-livestock interface areas in the region.

Limited studies exist on *T. gondii* infection in canids (domestic and wild), with an overall pooled prevalence of 69% (95% CI: 38–96%). This result is higher than the prevalence of 51.2.% reported in wild canids by Dubey et al. [71] and the global prevalence of 39.6% reported in foxes [72]. The studies in cattle were few and only done in South Africa and gave an overall pooled prevalence of 20% (95% CI: 5–39%), which is higher than the pooled prevalence of 16.3% (10.6–23.0%) from West Africa [73] and 12% (CI 8–17%) in the entire continent of Africa [1]. The estimated prevalence is, however, lower than the reported seroprevalence from Brazil and Sudan [74,75]. Studies have identified the consumption of raw or undercooked beef as a possible risk of toxoplasmosis transmission in humans [76,77].

Similarly, there is evidence of *T. gondii* infection in small ruminants (sheep and goats) [77], and the pooled prevalence of 11% (95% CI: 0–31%) recorded in this study is lower than that of 29.1% (15.6–44.8) in sheep and 18.1% (4.0–38%) in goats in West Africa [73] and sheep 26.1% (95% CI: 17.0–37.0%) and goats 22.9% (95% CI: 12.3–36.0%) in Africa [1]. Among livestock species, sheep constitutes an important source of animal protein as well as meat and milk from goats [78], whereas consumption of rare lamb and drinking of unpasteurized milk has been identified as risk factors in acute toxoplasmosis transmission in humans [77,79,80,81].

Studies reporting the seroprevalence of *T*. *gondii* in pigs in southern Africa emanated from South Africa and Zimbabwe, with an overall pooled prevalence of 13% (95% CI: 1–29%). This is similar to the prevalence reported in pigs from Europe [80] but lower than the prevalence reported in pigs from North America, South America, Asia [82], West Africa [73], Africa [1], and globally [82]. Pigs are among the popular food animals and have been reported as a source of human toxoplasmosis through ingestion of raw or undercooked pork [83]. *Toxoplasma gondii* infections in pigs are either acquired prenatally via transplacental transmission or postnatally via ingestion of oocysts from a contaminated environment [1]. Hence, indoor rearing of pigs is important to reduce the exposure of pigs to *T*. *gondii* infections from the contaminated environment [1,43,84].

The overall pooled prevalence of 22% (95% CI: 0–84%) of *T*. *gondii* seroprevalence from chickens and birds in southern African countries is lower than the estimated prevalence of anti-*T*. *gondii* antibody 22% (95% CI: 0–84%) reported in chickens in West Africa [73] and 37.41% (95% CI: 29.20–46.00%) from chickens in Africa [1]. Chicken meat is a key contributor to animal protein due to affordability and availability [85]; however, it also plays a major role in human toxoplasmosis transmission when the meat is consumed raw or undercooked [1]. The free-range chickens ingest *T*. *gondii* oocysts from the contaminated environment while foraging, thus acting as zoonotic agents of human toxoplasmosis. The role of birds, especially the birds of prey, in maintaining transmission between the sylvatic cycle and domestic cycle has also been documented [86].

The pooled seroprevalence of anti-*T*. *gondii* antibody from humans came from studies that focused mainly on immunocompetent individuals, HIV+ patients, and pregnant women [8,54,57,60,62,63] as well as a few studies on blood donors and children [56,61]. Overall, the pooled prevalence of 14% (95% CI: 5–25%) of *T*. *gondii* infection in humans from southern African countries was lower than the seroprevalence reported from a meta-analysis conducted on pregnant women in African regions, American regions, eastern Mediterranean regions, Europe, the South-East Asia region, globally [87], and in some North African countries (Tunisia, Egypt, and Morocco) [68]. However, this prevalence is greater than the seroprevalence reported from Western pacific region and the World Health Organization (WHO) regions of the world, 1.1% (0.8–1.4) [87]. Humans acquire *T*. *gondii* infections either through ingestion of oocysts from the contaminated environment [88,89], via tissue bradyzoites from consumption of raw or undercooked infected meat, transplacental transmission from mother to fetus [46,90], or organ transplants or blood transfusion [11,91]. Infections in immunocompetent individuals are not associated with critical symptoms compared to the immunosuppressed, particularly AIDS patients or newborns. Congenital transmission often results in clinical manifestations, such as encephalitis, pneumonia, and ophthalmologic disorders [1,68]. The seropositivity of *T*. *gondii* prevalence in the subjects in the reviewed articles suggests an active transmission of human toxoplasmosis in the region and requires intervention to prevent infection. Control and prevention measures include environmental control of feral cats, provision of veterinary care of domestic animals, adoption of personal hygiene, creating awareness of the risk associated with consumption of raw or undercooked meat, adequate screening of blood or organ donors, and adopting a national toxoplasmosis treatment scheme for pregnant women in the region [10,92].

Diagnostic tools used in the reviewed articles varied widely and ranged from MAT, LAT, IFAT, ELISA, DT, CF, Wolstenholme’s modification, and Sabin–Feldman dye test techniques to molecular approach. Studies have shown that different diagnostic techniques produce results that are heterogeneous [68]. For instance, the diagnostic performance of the MAT technique has been reported to be higher than that of ELISA [93]. In this study, the majority of articles adopted ELISA and IFAT to determine the seroprevalence of *T*. *gondii*. Although serological methods seem to lack sensitivity and specificity, they remain a standard tool for the qualitative detection of antibodies [68]. Studies that used LAT [3,56,60], histology [28,33,34], and molecular techniques [48,50,64] were few, while others used the combination of one or two of LAT, MAT, ELISA, IFAT, DT, CF, Wolstenholme’s modification, and Sabin–Feldman dye test techniques [30,32,35,41,42,54,59,93,94]. A recent study comparing three serological diagnostic tools showed that ELISA and IFAT had relatively higher sensitivity and specificity than MAT [95]. Additionally, ELISA and IFAT are less laborious and time-consuming than MAT [95]. As much as molecular tools are reliable diagnostic tools, they were used in only three studies. Molecular tools are ideal for determining the distribution of *T*. *gondii* in the environment (soil and water samples), and the few studies might have been attributed to the non-availability of this diagnostic facility or the lack of competent individuals for such analysis. The adoption of molecular methods (both PCR and more discriminatory and advanced molecular tools, such as PCR-RFLP markers and DNA sequencing) will be imperative in identifying the *T. gondii* strains infecting various hosts.

Generally, substantial heterogeneity existed between the studies reviewed and subgroups. This may be due to a range of factors, such as people’s varying hygiene practice levels, limited studies from some countries, varying diagnostic methods used, methods of rearing livestock animals, meat consumption pattern of studied individuals, or hostage.

## 4. Methods

### 4.1. Search Strategy

A systematic literature search was conducted in the following databases: Google Scholar, PubMed, EBSCO Host, and Science Direct using the following terms and Boolean operators (AND, OR): Toxoplasma AND Toxoplasmosis in southern Africa, Toxoplasma in cats AND southern Africa, Toxoplasma in livestock (sheep, goats, cattle) AND southern Africa, Toxoplasma in wildlife AND Southern Africa, Toxoplasma in felids, Toxoplasma in fowls AND Southern Africa, and Toxoplasma in humans AND southern Africa. The titles and abstracts of the search results were perused for the retrieval of relevant articles. References from selected articles were further used as a guide to other literature. The literature search was concluded in June 2021. Full-text articles were retrieved and managed in Endnote reference manager, version X7 (Clarivate Analytics, Philadelphia, PA, USA). This systematic review was performed following the PRISMA protocol (Reporting Items for Systematic Reviews and Meta-Analyses).

- Inclusion and exclusion criteria

An article was included in this study if it was published between 1955 and 2020 in a peer-reviewed journal and reported on (1) prevalence of *T*. *gondii* in cats and/or other animals and (2) *Toxoplasma* seroprevalence in humans in southern Africa. Dead links, duplicates, and grey pieces of literature were excluded during the literature review. The Preferred Reporting Items for Systematic Reviews and Meta-Analysis (PRISMA) used in this review is shown in (Figure 1).

- Data extraction and quality assessment

From each selected article, data on the study period, country of study, type of hosts, sample size, number of infected subjects/hosts, prevalence (%), and the diagnostic method(s) used were retrieved. Quality assessment of the identified articles was done as described by Munn et al. [96]. Quality assessment of each article was based on the following information: (1) relevance of research objective(s) to *Toxoplasma*, (2) prevalence of *Toxoplasma* as the main objective of the study, (3) study design was appropriately defined (case reports, cross-sectional), (4) samples randomly selected, (5) study subjects categorized by age/sex were relevant, (6) use of valid diagnostic methods in the study, (7) reliability of diagnostic methods, (8) representativeness of target sample to the general population, (9) description of the prevalence of *Toxoplasma* infection in the study community/animals, and (10) geographical location of *Toxoplasma* infection defined. The index score for each article was calculated by dividing the quality assessment of the study by ten. Detailed information about reasons for inclusion/exclusion and quality assessment is shown in Supplementary File S1.

### 4.2. Data Analysis

The extracted data from the search were entered in Microsoft Excel for analysis. The MetaXL (www.epigear.com accessed on 15 October 2021) was used to carry out a meta-analysis. An Inverse Heterogeneity (IVhet) model was used to compute the prevalence estimates with their 95% confidence intervals (CIs). The inverse variance statistic (I^2^ index) was used to quantify heterogeneity, and we tested for its significance using Cochran’s Q test. The I^2^ index was interpreted as no, low, moderate, or high heterogeneity if the value was 0%, ≤25%, 50%, or ≥75%, respectively. Forest plots were generated to show the prevalence of *Toxoplasma* among the study subjects. Furthermore, subgroup analysis was carried out to assess the mean pooled prevalence estimates according to host types and regions within southern Africa. The risk of publication bias was assessed using the Luis Furuya–Kanamori (LFK) index and funnel plot [97]. The symmetry of the Doi plots was determined using the LFK index and a value within the range of ±1 was considered as symmetrical and classified as the absence of publication bias, while an LFK value within the range of ±2 was considered as minor asymmetry with slight publication bias, and an LFK value outside the range of ±2 was considered as major asymmetry and high publication bias [97].

## 5. Conclusions and Recommendation

This study showed that there are limited studies on *T*. *gondii* in humans and animals in southern Africa. Considering the limited information on the prevalence of *T. gondii* in southern African countries, more studies targeting the epidemiology of this parasite in the environment (soil and water), vegetable, food animals, wild animals, and humans (children, pregnant women, immunocompromised, and healthy people) must be conducted to better understand the transmission dynamics in the region. Additionally, there is a need to establish a surveillance system at the wild animals-livestock interface for monitoring transmission between livestock, wildlife, and humans. Furthermore, emphasis should be focused on health education and the preventive measures of toxoplasmosis, which include adequate cooking of meat, washing of fruits and vegetables before eating, and provision of potable water.

## Figures and Tables

**Table 1:** Studies on the prevalence of *Toxoplasma gondii* in domestic canids and felids canids in southern African countries from 1961 to 2019.

Study Country	Host Species	*n*	Np	(%)	Diagnostic Test	Study Period	Quality Index Score	References
South Africa	Dogs	7	7	100	Histology	1955–1961	0.7	Smit 1961 [28]
South Africa	Cats	102	22	21.6	ELISA	2012	0.6	Lobetti and Lappin, 2012 [29]
South Africa	Cats	1	1	100	Histology and PCR	2012	0.9	Nagel, Williams, and Schoeman, 2013 [30]
South Africa	Cats	159	83	52.2	IFAT	2013–2014	0.8	Kenneth Hammond-Aryeea et al., 2015 [31]
Angola	Cats	102	4	3.9	MAT	2014–2016	0.7	Lopes et al., 2017 [32]
South Africa	Cats	109	35	32.1	LAT	2016	0.9	Tagwireyi et al., 2019 [3]

*n*, sample size; Np, number positive.

**Table 2:** Studies on the prevalence of *Toxoplasma gondii* in wildlife in southern African countries from 1966 to 2020.

Study Area	Host Species	*n*	Np	(%)	Diagnostic Test	Study Period	Quality Index Score	References
South Africa	Ferrets	7	4	42.9	Histology	1966	0.5	Bigalke et al., 1966 [33]
South Africa	Chinchilla	5	5	100	Histology	1966	0.5	Du Plessis et al., 1967 [34]
South Africa	Baboons	90	30	11.7	IFAT, CF, Wolstenholme’s modification, Sabin–Feldman dye test	1969–1971	0.8	Mc Connell et al., 1973 [35]
Namibia	Lions	66	65	98	Indirect Immunofluorescence Assay	1989–1991	0.6	Spencer 1993 [36]
South Africa	Lions	18	18	100	IFAT	1984–1996	0.8	Cheadle, Spencer, and Blagburn, 1999 [37]a
South Africa	Leopard	2	2	100	IFAT	1984–1996	0.8	Cheadle, Spencer, and Blagburn, 1999 [37]b
South Africa	Lions	5	5	100	IFAT	1984–1996	0.8	Cheadle, Spencer, and Blagburn, 1999 [37]c
South Africa	Lions	3	3	100	IFAT	1984–1996	0.8	Cheadle, Spencer, and Blagburn, 1999 [37]d
Botswana	Leopard	2	1	50	IFAT	1984–1996	0.8	Cheadle, Spencer, and Blagburn, 1999 [37]e
Botswana	Cheetah	1	0	0	IFAT	1984–1996	0.8	Cheadle, Spencer, and Blagburn, 1999 [37]f
Namibia	Lions	1	1	100	IFAT	1984–1996	0.8	Cheadle, Spencer, and Blagburn, 1999 [37]g
Namibia	Cheetah	6	2	33.3	IFAT	1984–1996	0.8	Cheadle, Spencer, and Blagburn, 1999 [37]h
South Africa	Cheetah	16	8	50	IFAT	1984–1996	0.8	Cheadle, Spencer, and Blagburn, 1999 [37]i
South Africa	Lions	5	5	100	IFAT	1984–1996	0.8	Cheadle, Spencer, and Blagburn, 1999 [37]j
South Africa	Lions	9	5	55.6	IFAT	1984–1996	0.8	Cheadle, Spencer, and Blagburn, 1999 [37]k
Botswana	Lions	53	49	92	IFAT	2002	0.5	Penzhorn et al., 2002 [38]a
Zimbabwe	Lions	21	21	100	IFAT	2002	0.5	Penzhorn et al., 2002 [38]b
South Africa	Lions	12	12	100	IFAT	2002	0.5	Penzhorn et al., 2002 [38]c
South Africa	Lions	30	30	100	IFAT	2002	0.5	Penzhorn et al., 2002 [38]d
Botswana	Leopard	1	1	100	IFAT	2002	0.5	Penzhorn et al., 2002 [38]e
South Africa	Leopard	7	6	86	IFAT	2002	0.5	Penzhorn et al., 2002 [38]f
South Africa	Caracal	29	24	83	IFAT	2014–2017	0.9	Serieys et al., 2019 [39]
Namibia	African Lion	59	55	93.2	ELISA	2002–2015	0.6	Seltmann et al., 2020 [40]a
Namibia	Brown hyena	19	12	92.3	ELISA	2002–2015	0.6	Seltmann et al., 2020 [40]b
Namibia	Caracal	15	10	66.7	ELISA	2002–2015	0.6	Seltmann et al., 2020 [40]c
Namibia	Cheetah	250	131	52.4	ELISA	2002–2015	0.6	Seltmann et al., 2020 [40]d
Namibia	Leopard	58	47	81	ELISA	2002–2015	0.6	Seltmann et al., 2020 [40]e
Namibia	Spotted hyena	11	10	90.9	ELISA	2002–2015	0.6	Seltmann et al., 2020 [40]f
Namibia	African wild dog	7	5	71.4	ELISA	2002–2015	0.6	Seltmann et al., 2020 [40]g
Namibia	Bat eared fox	4	1	25	ELISA	2002–2015	0.6	Seltmann et al., 2020 [40]h
Namibia	Black backed jackal	39	26	66.7	ELISA	2002–2015	0.6	Seltmann et al., 2020 [40]i
Namibia	Honey badger	10	7	70	ELISA	2002–2015	0.6	Seltmann et al., 2020 [40]j
Namibia	Blue-wildebeest	20	2	10	ELISA	2002–2015	0.6	Seltmann et al., 2020 [40]k
Namibia	Springbok	20	0	0	ELISA	2002–2015	0.6	Seltmann et al., 2020 [40]l

*n*, sample size; Np, number positive. The different letters are there to show that the hosts are different and so are the citations.

**Table 3:** Studies on the prevalence of *Toxoplasma gondii* in livestock in southern African countries from 1992 to 2020.

Study Country	Host Species	*n*	Np	(%)	Diagnostic Test	Study Period	Quality Index Score	References
Zimbabwe	Sheep	216	13	8.8	LAT and ELISA	1992	0.7	Pandey and Van Knapen, 1992 [41]a
Zimbabwe	Goats	156	7	7.1	LAT and ELISA	1992	0.7	Pandey and Van Knapen, 1992 [41]b
Zimbabwe	Pigs	311	10	4.2	LAT and ELISA	1992	0.7	Pandey and Van Knapen, 1992 [41]c
Zimbabwe	Pigs	97	9	9.3	MAT	1995	0.7	Hove and Dubey 1999 [42]
Zimbabwe	Pigs	238	47	19.75	IFAT and ELISA	2000–2002	0.8	Hove et al., 2005a [43]a
Zimbabwe	Pigs	70	25	35.71	IFAT and ELISA	2000–2002	0.8	Hove et al., 2005a [43]b
South Africa	Sheep	600	26	4.3	ELISA	2007	0.9	Abu Samraa et al., 2007 [44]
South Africa	Cattle	178	37	20.8	ELISA	2012	0.8	Ndou et al., 2013 [45]
South Africa	Sheep	292	23	7.9	ELISA	2014	0.9	Hammond-Aryee et al., 2015 [46]
South Africa	Sheep	121	78	64.5	LAT	2016	0.9	Tagwireyi et al., 2019 [3]a
South Africa	Goats	128	69	53.9	LAT	2016	0.9	Tagwireyi et al., 2019 [3]b
South Africa	Pigs	106	36	34	LAT	2016	0.9	Tagwireyi et al., 2019 [3]c
South Africa	Cattle	184	60	32.6	ELISA	2013	0.8	Adesiyun et al., 2020 [47]
South Africa	Cattle	109	5	4.6	PCR	2019	0.8	Mofokeng 2020 [48]

*n*, sample size; Np, number positive. The different letters are there to show that the hosts are different and so are the citations.

**Table 4:** Studies on the prevalence of *Toxoplasma gondii* in fowls (chicken and birds) in southern African countries from 2001 to 2019.

Study Area	Host Species	*n*	Np	(%)	Diagnostic Test	Study Period	Quality Index Score	References
Botswana	Pigeons	16	16	100	Indirect Haemaglutination Test (IHT)	2001	0.4	Mushi et al., 2001 [49]
South Africa	Birds	110	3	2.7	PCR	2014–2015	0.7	Lukášová et al., 2018 [50]
South Africa	Chickens	137	46	33.6	LAT	2016	0.9	Tagwireyi et al., 2019 [3]d

*n*, sample size; Np, number positive. The different letters are there to show that the hosts are different and so are the citations.

**Table 5:** Studies on seroprevalence of toxoplasmosis reported in humans in southern African countries from 1974 to 2017.

Study Area	Human Description	*n*	Np	(%)	Diagnostic Test	Study Period	Quality Index Score	References
South Africa	People from different ethnic groups	806	296	37	IFAT	1974	0.8	Masons et al., 1974 [51]
South Africa	Reproductive age women	600	3	0.5	IFAT	1975	0.8	Brink et al., 1975 [52]
Southern Africa	Blood donors from diverse ethnic groups	3379	665	20	IFAT	1978	0.8	Jacobs and Mason 1978 [53]
Zambia	HIV-positive individuals	187	8	4.3	LAT and DT	1991	0.8	Zumla et al., 1991 [54]a
Zambia	HIV-negative individuals	189	20	10.6	LAT and DT	1991	0.8	Zumla et al., 1991 [54]b
South Africa	HIV-positive individuals	307	25	8	ELISA	2007	0.9	Hari et al., 2007 [55]
Swaziland	Apparently healthy children	113	5	4.4	LAT	2009	0.8	Liao et al., 2009 [56]
South Africa	HIV-positive individuals	160	29	18.1	ELISA	2007–2008	0.8	Bessong and Mathomu 2010 [57]
Mozambique	HIV-positive patients	150	28	18.7	ELISA	2010	0.7	Sitoe et al., 2010 [58]
South Africa	Immunocompetent individuals	497	32	6.4	Pastorex Toxo latex particle agglutination test and BioMèrieux ToxoScreen DA test	2011	0.8	Kistiah 2011 [59]a
South Africa	HIV-negative patients	376	48	12.8	Pastorex Toxo latex particle agglutination test and BioMèrieux ToxoScreen DA test	2011	0.8	Kistiah 2011 [59]b
South Africa	HIV-positive patients	376	37	9.8	Pastorex Toxo latex particle agglutination test and BioMèrieux ToxoScreen DA test	2011	0.8	Kistiah 2011 [59]c
Mozambique	HIV-positive men	200	20	39.3	LAT	2010	0.7	Domingos et al., 2013 [60]a
Mozambique	HIV-positive women	200	25	50.9	LAT	2010	0.7	Domingos et al., 2013 [60]b
Namibia	Blood donor	312	4	1.3	ELISA	2011–2012	0.8	van der Colf, Noden, Wilkinson, and Chipare, 2014 [61]
Zambia	Pregnant women	411	24	5.9	OnSite Toxo IgG/IgM Combo Rapid test	2015	0.8	Frimpong et al., 2017 [19]
South Africa	HIV-positive individuals	161	61	38	ELISA	2012–2013	0.7	Ngobeni and Samie, 2017 [62]a
South Africa	HIV-negative individuals	161	27	16.7	ELISA	2012–2013	0.7	Ngobeni and Samie, 2017 [62]b
Namibia	Pregnant women	344	9	2.61	ELISA	2016	0.9	Van der Colf et al., 2020 [63]

*n*, sample size; Np number positive. The different letters are there to show that the hosts are different and so are the citations.

## Data Availability

All detailed information about reasons for inclusion/exclusion and quality assessment as well as Supplementary Materials are available on request.

## Supplementary Materials

The following are available online at https://www.mdpi.com/article/10.3390/pathogens11020183/s1, Supplementary File S1: Quality assessment checklist for the study.
