# Supplementary material for: Toxoplasma gondii Infections in Animals and Humans in Southern Africa: A Systematic Review and Meta-Analysis
Source: Pathogens. 2022 Jan 28;11(2):183. doi: 10.3390/pathogens11020183 (PMC8880191; doi:10.3390/pathogens11020183)
Supplement: Supplementary file 1 [file pathogens-11-00183-s001.zip › pathogens-1517976-supplementary.pdf]

Supplementary File S1: **Quality assessment checklist**

The following items were examined and given a score based on a simple scale system (1 for "yes", 0 for "no").

1. Was the research objective relevant to the review?

2. Was toxoplasmosis the main objective of the study?

3. Were the subjects of study categorised by age/sex and were the age categories clearly defined?

4. Were the methods of diagnosis confirmative for the presence of toxoplasmosis in the subject of study either directly on subjects or inferred from immunological/molecular results?

5. Were number and percentage of positive subjects clearly described?

6. Were geographical region of toxoplasmosis clearly reported?

7. Were the target sample a close representation of the general population?

8. Was there a form of random selection used to select the samples?

9. Was a minimum sample size calculated?

10. Was sampling period for toxoplasmosis clearly stated?

The quality index score for each study was calculated by dividing the study quality score by 10.
